# Guselkumab in Inflammatory Bowel Disease: Mechanism, Clinical Efficacy, Safety, and Treatment Positioning

**DOI:** 10.3390/jcm15176667

**Published:** 2026-08-28

**Authors:** Laura White, Esha Sharma, Jimmy Limdi, Shahida Din, Kamal Patel, Sailish Honap

**Affiliations:** 1 Department of Gastroenterology, London Northwest University Healthcare Trust, London SW17 0QT, UK; laura.white18@nhs.net; 2 Department of Gastroenterology, Guy’s and St Thomas’ NHS Foundation Trust, London SE1 7EH, UK; 3 Manchester Academic Health Sciences Faculty of Biology, Medicine & Health, University of Manchester, Manchester BL9 7TD, UK; 4 IBD Section, Department of Gastroenterology, Northern Care Alliance NHS Foundation Trust, Manchester M6 8HD, UK; 5 Edinburgh Inflammatory Bowel Diseases Unit, NHS Lothian, Edinburgh EH4 2XU, UK; 6 Institute of Genetics and Cancer, University of Edinburgh, Edinburgh EH4 2XU, UK; 7 Department of Gastroenterology, St George’s University Hospital NHS Foundation Trust, London SW17 0QT, UK; 8 Department of Immunobiology, School of Immunology and Microbial Sciences, King’s College London, London SE1 9RT, UK; 9 Institute of Infection and Immunology, City St George’s Hospital, University of London, London SW17 0RE, UK

**Keywords:** Crohn’s disease, guselkumab, inflammatory bowel disease, interleukin-23, IL-23p19 inhibitor, ulcerative colitis

## Abstract

Guselkumab is a monoclonal antibody directed against the p19 subunit of interleukin-23 (IL-23), thereby preventing IL-23 binding to the IL-23 receptor. IL-23 is a key driver of immune dysregulation and chronic inflammation in inflammatory bowel disease (IBD), making selective IL-23 inhibition an established therapeutic strategy in both Crohn’s disease and ulcerative colitis. Guselkumab is the most recently approved IL-23p19 inhibitor for the treatment of both major forms of IBD. It is the first IL-23p19 inhibitor to offer a fully subcutaneous induction regimen, and preliminary phase III data has suggested potential benefits for patients with perianal Crohn’s disease. In registrational trials, there were significant improvements across multiple clinical and endoscopic endpoints in comparison to ustekinumab, although these trials were not powered for formal superiority testing. This narrative review summarises the biological rationale for IL-23 blockade, reviews the available clinical evidence, discusses practical considerations for its use, and considers its place in the landscape of IBD treatments.

## 1. Introduction

IBD, comprising Crohn’s disease (CD) and ulcerative colitis (UC), is characterised by chronic intestinal inflammation arising from a complex interplay between genetic susceptibility, environmental exposures, the gut microbiota, and dysregulated immune responses [1,2,3,4]. The therapeutic landscape for inflammatory bowel disease (IBD) has evolved rapidly over the past decade. Excluding immunomodulators, 15 advanced therapies are approved by the US Food and Drug Administration (FDA) and 14 by the European Medicines Agency (EMA) for the treatment of moderate-to-severe IBD, spanning multiple therapeutic classes that target distinct inflammatory pathways [4]. These advances have expanded treatment options considerably and shifted clinical practice towards earlier use of advanced therapies to alter the natural course of disease.

Increasing evidence supports early intervention with advanced therapies using a ‘top-down’ treatment strategy to reduce disease progression, prevent complications, and improve quality of life [5]. Despite these advances, approximately 30% of patients fail to respond to induction therapy, while up to half of initial responders subsequently lose response, highlighting the ongoing need for therapies with improved efficacy, durability, and safety [2,6]. Furthermore, therapeutic options for fistulising perianal Crohn’s disease remain limited, with durable fistula healing achieved in only a minority of patients despite combined medical and surgical management [7].

Among the inflammatory pathways implicated in IBD, interleukin (IL)-23 has emerged as a central regulator of chronic intestinal inflammation. IL-23 and IL-12 belong to the IL-12 cytokine family and share a common p40 subunit, whereas the p19 subunit is unique to IL-23 [8]. Ustekinumab, which targets the shared p40 subunit, inhibits both IL-12 and IL-23 and is an established treatment for IBD [9,10]. However, accumulating evidence suggests that IL-23 is the principal pathogenic cytokine driving intestinal inflammation, whereas IL-12 may exert protective or regulatory functions in certain settings [11]. This has provided the biological rationale for selectively targeting the IL-23-specific p19 subunit, leading to the development of the IL-23p19 inhibitor class [12].

Guselkumab (Tremfya; Johnson & Johnson, New Brunswick, NJ, USA) is the most recently approved IL-23p19 inhibitor for the treatment of UC and CD, joining risankizumab (Skyrizi; AbbVie, North Chicago, IL, USA) and mirikizumab (Omvoh; Eli Lilly, Indianapolis, IN, USA) [13]. Following the phase III QUASAR programme, guselkumab received FDA approval for UC in September 2024 [14]. Approval for CD followed in March 2025 based on the GALAXI programme [15], with subsequent approval by the UK Medicines and Healthcare products Regulatory Agency (MHRA) in May 2025 [15,16].

This narrative review summarises key efficacy and safety data for guselkumab from the clinical trial programmes, highlights practical implications for its use for the treatment of moderate-to-severe UC and CD, and reviews available data to guide positioning of guselkumab in the IBD therapeutic landscape.

## 2. Methods

A comprehensive literature search was performed in April 2026 using MEDLINE via PubMed using the search strategy “guselkumab” AND (“Crohn’s disease” OR “ulcerative colitis” OR “inflammatory bowel disease”). A language restriction of articles published in English was also included in the search. Additional relevant publications were identified through manual screening of reference lists. Three of the listed authors were associated with evidence selection (LW, ES and SH).

The results of full peer-reviewed publications were included as well as wider evidence sources such as registered clinical trials and, where relevant, conference abstracts. Any results presented in the absence of full peer-reviewed publication were regarded as preliminary.

This search was updated with a full final search on 5 July 2026 to include the most recent publications; in particular, this was relevant to recently published abstracts from major international conferences as well as newly registered clinical trials.

## 3. Pharmacology of Guselkumab

### 3.1. Development of Guselkumab (Formerly CNTO-1959)

Guselkumab (formerly CNTO-1959; Tremfya^®^, Johnson & Johnson, New Brunswick, NJ, USA) is a fully human IgG1λ monoclonal antibody developed through a collaboration between MorphoSys AG and Centocor Inc., Malvern, PA, USA (now Janssen Research & Development, LLC, Raritan, NJ, USA) using the Human Combinatorial Antibody Library (HuCAL^®^, Bio-Rad Laboratories, Hercules, CA, USA) platform. Its development coincided with increasing recognition of the central role of the IL-23/Th17 pathway in chronic immune-mediated inflammatory diseases [17].

Unlike ustekinumab, which targets the p40 subunit shared by IL-12 and IL-23, guselkumab was designed to selectively bind the p19 subunit unique to IL-23, thereby inhibiting IL-23 signalling while preserving IL-12-dependent immune pathways [18]. This selective approach was based on emerging evidence that IL-23 is the principal pathogenic cytokine driving chronic inflammation, whereas IL-12 may exert protective or immunoregulatory functions in certain settings. Early preclinical and clinical studies demonstrated substantial efficacy in plaque psoriasis, leading to regulatory approval and establishing proof-of-concept for selective IL-23 inhibition [19]. Subsequent clinical development expanded to psoriatic arthritis and, more recently, ulcerative colitis and Crohn’s disease following the recognition of IL-23 as a central driver of intestinal inflammation [14].

### 3.2. Pharmacological Properties of Guselkumab

**Figure 1:**
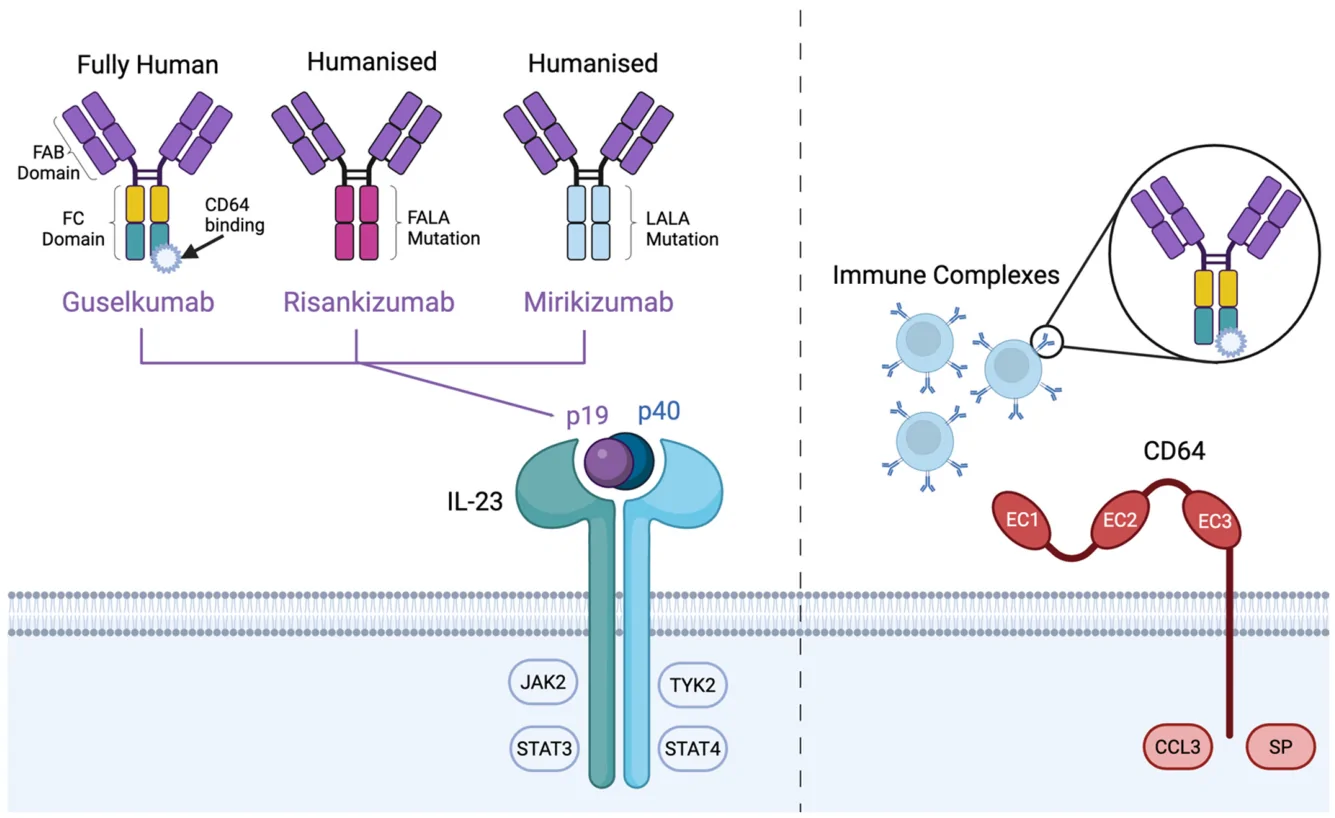
Mechanism of action and structural characteristics of IL-23p19 inhibitors approved for inflammatory bowel disease. Guselkumab, risankizumab, and mirikizumab are monoclonal antibodies that selectively bind the p19 subunit of interleukin (IL)-23, preventing activation of the IL-23 receptor and downstream signalling. Unlike ustekinumab, selective p19 inhibition preserves IL-12 signalling through the shared p40 subunit. This selective approach targets IL-23 as the principal pathogenic cytokine driving chronic inflammation, whereas IL-12 may exert protective or immunoregulatory functions in certain settings. Guselkumab is a fully human monoclonal antibody, whereas risankizumab and mirikizumab are humanised antibodies. Risankizumab and mirikizumab incorporate Fc mutations (FALA and LALA, respectively) that minimise Fc-mediated effector functions. In contrast, guselkumab retains Fcγ receptor (CD64) binding activity, enabling engagement with CD64-expressing myeloid cells following formation of IL-23–guselkumab immune complexes. Preclinical studies suggest this interaction may enhance neutralisation of IL-23 at its cellular source through CD64-mediated internalisation of IL-23–guselkumab complexes. However, the clinical relevance of these findings remains uncertain. Figure created using https://BioRender.com.

Guselkumab is a fully human IgG1λ monoclonal antibody that selectively binds the p19 subunit of IL-23 with high affinity, thereby preventing activation of the IL-23 receptor and downstream JAK2/TYK2 signalling (Figure 1). Unlike risankizumab and mirikizumab, guselkumab retains an unmodified Fc domain capable of binding the high-affinity Fcγ receptor I (CD64) (Figure 1). Preclinical studies suggest that this facilitates binding to CD64-expressing IL-23-producing myeloid cells, internalisation of IL-23–guselkumab immune complexes, and potentially enhancing neutralisation of IL-23 at its cellular source [20]. However, the clinical relevance of these findings remains uncertain.

Immunogenicity was low across the guselkumab clinical development programme. Across phase III studies, anti-drug antibodies developed in approximately 9–11% of participants, with neutralising antibodies detected in around 1% [21]. Long-term extension studies similarly demonstrated low rates of immunogenicity, with no clear association between anti-drug antibody development and reduced efficacy or new safety signals [22]. Compared with other IL-23p19 inhibitors, the reported immunogenicity for guselkumab appears lower than that of mirikizumab and slightly higher than that of risankizumab, although clinically meaningful consequences remain uncommon.

### 3.3. Pharmacokinetic Profile

Beyond its pharmacodynamic properties, understanding the pharmacokinetic profile of guselkumab is important for interpreting its dosing strategies and clinical use. In phase II clinical trials, the IV induction dose of 200 mg at Weeks 0, 4, and 8 demonstrated near maximal efficacy with no additional benefit from larger doses [23,24]. Following subcutaneous administration, the absolute bioavailability of guselkumab is approximately 50% [25]. Consequently, the subcutaneous induction regimen evaluated in the phase III programme used double the intravenous induction dose to achieve comparable overall drug exposure. Although subcutaneous induction produced lower peak and higher trough serum concentrations than intravenous administration did, average drug exposure over the induction period was similar, with serum concentrations reaching steady state by Week 24 irrespective of induction route [21,26,27]. These findings support the use of either intravenous or fully subcutaneous induction regimens for both UC and CD (Table 1).

The elimination half-life of guselkumab is approximately 17 days in patients with UC and CD [25]. This is longer than that of mirikizumab (approximately 9.3 days) [28] and shorter than that of risankizumab (21–29 days) [29]. Population pharmacokinetic analyses demonstrated that concomitant immunomodulators, oral corticosteroids, age, renal function, and hepatic function had no clinically meaningful effects on drug clearance, indicating that dose adjustment is generally unnecessary [25]. A further population pharmacokinetic analysis also demonstrated that body weight, serum albumin, CRP, age, sex, and prior biologic failure status also had no clinically meaningful effects on drug clearance, confirming that dose adjustment on this basis is also unnecessary [30].

Exposure–response analyses demonstrated comparable efficacy of intravenous and subcutaneous induction across body weight and BMI categories in both UC and CD. These findings suggest that obesity does not significantly influence clinical response and support fixed-dose induction without weight-based adjustment [31]. Therapeutic drug monitoring data for guselkumab in IBD are not available with current dosing regimens achieving maximal efficacy. There is currently insufficient evidence to support therapeutic drug monitoring with guselkumab.

## 4. Efficacy and Safety from the Clinical Trial Programmes

### 4.1. Ulcerative Colitis

The efficacy and safety of guselkumab for moderate-to-severe UC were established in the phase III QUASAR and ASTRO clinical trial programmes (Table 2). QUASAR used a responder re-randomised design to evaluate intravenous (IV) induction followed by maintenance therapy, whereas ASTRO adopted a treat-through design to evaluate a fully subcutaneous (SC) induction and maintenance strategy [14,32].

In QUASAR, guselkumab demonstrated superiority over the placebo for the primary endpoint of clinical remission as early as after the first of the three required IV induction doses (22.6% vs. 7.9%; *p* ≤ 0.001), with significant improvements across all major secondary endpoints, including clinical response, symptomatic remission, endoscopic improvement, and histological–endoscopic mucosal improvement at Week 12 [14]. During maintenance, both approved dosing regimens remained superior to the placebo, with clinical remission achieved in 45.2% of participants receiving 100 mg every 8 weeks and 50% receiving 200 mg every 4 weeks compared with 18.9% receiving the placebo. No new safety signals were identified, with rates of serious adverse events comparable to those of participants receiving the placebo [14].

ASTRO subsequently demonstrated that a fully SC induction strategy achieved similarly robust efficacy, with clinical remission observed in 28% of participants at Week 12 compared with 6% receiving the placebo (*p* ≤ 0.001) and sustained efficacy through Week 24 across both maintenance regimens [32]. These findings established the fully SC induction regimen as an effective alternative to IV induction, providing greater flexibility for patients and clinicians without compromising efficacy or safety.

### 4.2. Crohn’s Disease

The efficacy and safety of guselkumab for moderate-to-severe CD were established through the phase III GALAXI-2, GALAXI-3, and GRAVITI clinical trial programmes (Table 3). GALAXI-2 and GALAXI-3 were identically designed, randomised, double-blind, treat-through trials evaluating intravenous (IV) induction followed by maintenance therapy with two approved guselkumab dosing regimens. Both studies included placebo and ustekinumab comparator arms, although they were not powered to formally compare efficacy between guselkumab and ustekinumab [15]. GRAVITI subsequently evaluated a fully subcutaneous (SC) induction and maintenance strategy using the same approved maintenance regimens [21].

Across both GALAXI trials, guselkumab demonstrated superiority over the placebo for clinical response at Week 12 and for both co-primary endpoints of clinical remission and endoscopic response at Week 48 [15]. Clinical remission at Week 48 was achieved in 55% and 49% of participants receiving guselkumab 200 mg every 4 weeks and 100 mg every 8 weeks, respectively, in GALAXI-2, compared with 12% receiving the placebo. Corresponding rates in GALAXI-3 were 48%, 47%, and 13%, respectively. Endoscopic response rates were similarly superior for both guselkumab regimens across both studies. Guselkumab was well tolerated, with no new safety signals identified and rates of serious adverse events comparable to those of participants receiving the placebo [15].

Although the GALAXI programme was not designed or powered to demonstrate superiority over ustekinumab, pooled analyses suggested greater improvements across several clinical and endoscopic outcomes, including endoscopic response, endoscopic remission, and deep remission at Week 48. These findings are consistent with those of the SEQUENCE trial and further support the concept that selective IL-23p19 inhibition may offer greater efficacy than dual IL-12/23 blockade in Crohn’s disease [33].

GRAVITI subsequently demonstrated that a fully SC induction regimen achieved robust efficacy comparable to IV induction, with clinical remission at Week 48 achieved in 60% of participants receiving 100 mg every 8 weeks and 66.1% receiving 200 mg every 4 weeks compared with 17.1% receiving the placebo [21]. Endoscopic response rates were likewise significantly higher than those for the placebo, with no new safety concerns identified. Together with the ASTRO programme for UC, these findings establish guselkumab as the first IL-23p19 inhibitor with an effective fully SC induction regimen.

### 4.3. Clinical Trials Relating to Specific Clinical Circumstances

Extraintestinal manifestations (EIMs) can result in significant morbidity in people living with IBD and frequently influence treatment selection. Guselkumab is currently licensed for moderate-to-severe plaque psoriasis and active psoriatic arthritis. In patients with IBD and confirmed psoriatic arthritis, guselkumab may represent an attractive option because it could potentially address intestinal, cutaneous and peripheral joint manifestations with a single treatment. It is important to highlight that these conditions are distinct from the wider subpopulation of IBD patients with EIMs, such as axial manifestations, in which the evidence base is not well defined. In a pooled analysis of the phase III GALAXI-2 and GALAXI-3 studies, guselkumab was associated with higher rates of EIM resolution than the placebo at Week 12 (59.2% vs. 42.9%), with the greatest improvements observed in arthritis/arthralgia and erythema nodosum/pyoderma gangrenosum, although the presence of EIMs was patient self-reported without use of validated rheumatological outcome measures. Benefits were maintained through Week 48, and fewer patients developed new-onset EIMs during follow-up, suggesting that guselkumab may have a role in both the treatment and prevention of EIMs. It is important to note that these results are only available as a published conference abstract and should be regarded as preliminary [34].

**Figure 2:**
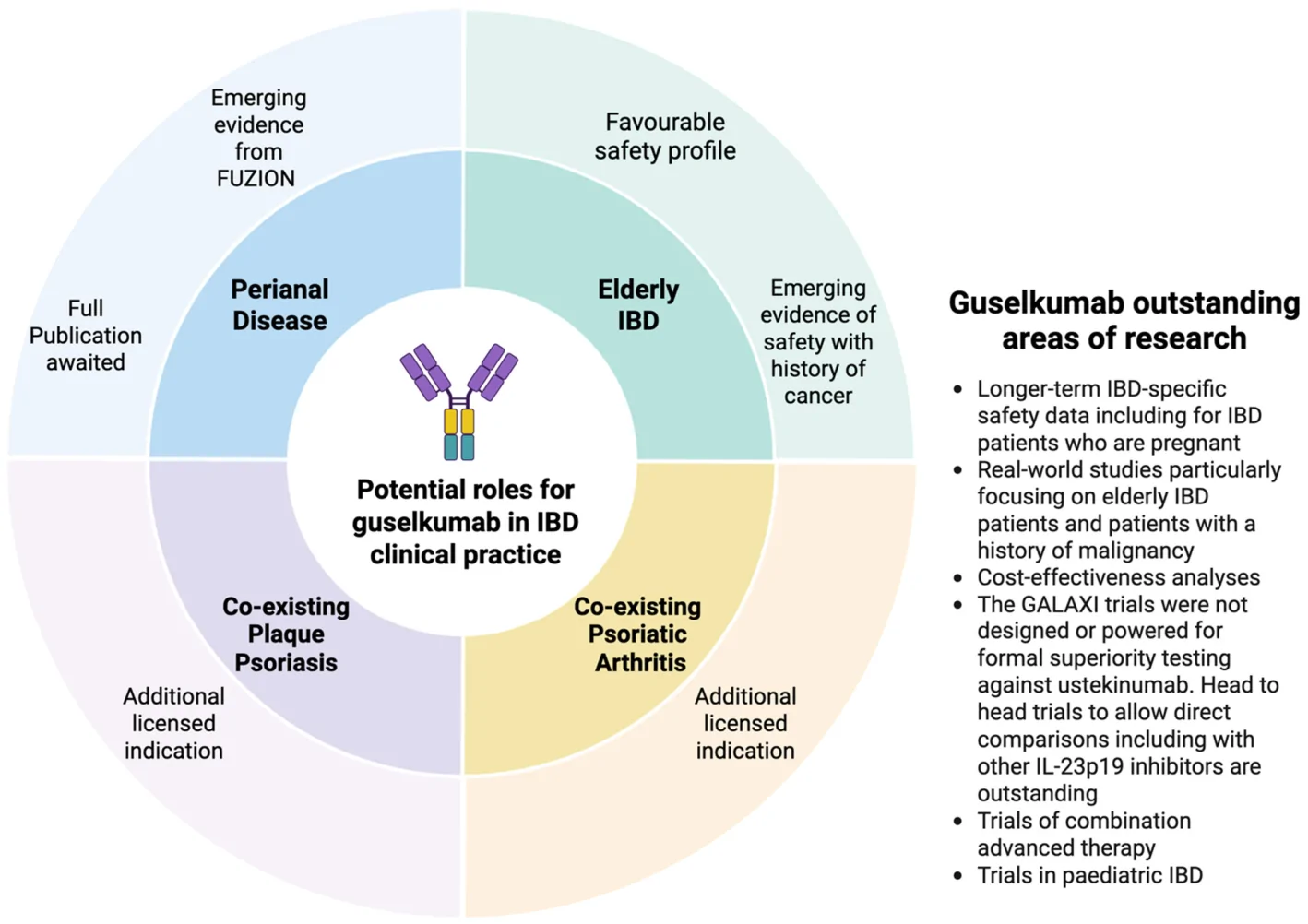
Summary of the potential key roles for guselkumab in IBD clinical practice. Examples of outstanding areas of research which will also influence the roles for guselkumab in IBD clinical practice. Created using https://BioRender.com.

Perianal fistulising CD remains one of the most challenging manifestations of IBD, with substantial effects on quality of life and limited effective medical treatment options. FUZION is the first phase III randomised, placebo-controlled trial specifically designed to evaluate guselkumab in perianal fistulising Crohn’s disease. The primary endpoint was combined fistula remission at Week 24, incorporating both clinical closure of all treated external fistula openings and radiological healing confirmed by central magnetic resonance imaging review. Preliminary results demonstrated combined fistula remission in 28.3% and 27.0% of participants receiving guselkumab 100 mg every 8 weeks and 200 mg every 4 weeks, respectively, compared with 10.3% receiving the placebo. Clinical fistula response (≥50% reduction from baseline in the number of open or draining perianal fistulas) was observed in 32.7–35.7% of guselkumab-treated participants versus 13.8% receiving the placebo, with separation from the placebo evident as early as Week 4 [35,36]. Although peer-reviewed publication of the full study is awaited, these findings suggest that guselkumab may represent a promising therapeutic option for this major unmet clinical need (Figure 2).

The efficacy and safety of guselkumab in children and older adults with IBD remain areas of active investigation. Dedicated paediatric phase III studies are ongoing [37]. Although population pharmacokinetic analyses indicate that age does not meaningfully influence drug clearance [25], older adults were under-represented in the pivotal IBD trials, comprising only 3.8% of participants with CD and 6.1% of those with UC. Real-world studies in psoriasis have demonstrated favourable effectiveness and tolerability in adults aged 65 years and older, but disease-specific data for IBD remain limited. Given that adults aged 60 years and older now represent one of the fastest-growing populations living with IBD in the UK, dedicated studies in this group are needed to better define the effectiveness and safety of guselkumab in routine clinical practice [38].

## 5. Safety

Across the guselkumab IBD clinical trial programme, the safety profile was broadly consistent with those observed in other approved indications, with no new safety signals identified. Rates of serious adverse events were generally comparable between the guselkumab and placebo arms in the pivotal UC and CD trials (Table 2 and Table 3). In addition, a recent systematic review and network meta-analysis of 18 clinical trials in Crohn’s disease (*n* = 5561) reported a lower risk of serious adverse events for IL-23p19 inhibitors as a class compared with placebo [39].

The available IBD trial data for guselkumab are limited by follow-up of up to 48 weeks. Longer-term safety data for guselkumab is therefore largely derived from psoriasis and psoriatic arthritis studies. In an integrated analysis of 2819 guselkumab-treated patients followed for up to 5 years, rates of serious infection, major adverse cardiovascular events, and malignancy did not increase over time [40]. It is important to emphasise that extrapolation to IBD should be made cautiously given differences in the disease population, comorbidity burden, concomitant corticosteroid exposure, and prior advanced therapy use.

Older adults were under-represented in the pivotal IBD trials, comprising only 3.8% of participants with Crohn’s disease and 6.1% of those with ulcerative colitis. Data for patients with previous malignancy also remain limited. Although there is no published evidence directly obtained from patients with IBD regarding the use of guselkumab in these settings except in singlVOYAGE 1 and 2, which evaluated guselkumab in moderate-to-severe psoriasis (*n* = 1721), 18 participants had a history of malignancy; one recurrence was reported, and overall malignancy rates at 5 years were comparable with those expected in the psoriasis population [41]. Dedicated real-world IBD studies with long-term follow-up will be required to define safety in these higher-risk groups (Figure 2).

Evidence regarding guselkumab exposure during pregnancy also remains limited. A pooled Johnson & Johnson safety analysis across approved indications included 1126 pregnancy outcomes and reported rates of live birth, spontaneous abortion, and congenital anomalies comparable with background population rates [42]. As an IgG1 monoclonal antibody, guselkumab would be expected to undergo placental transfer, particularly in the second and third trimesters. Nevertheless, a recent global consensus statement on pregnancy in IBD recommended that patients established on guselkumab may continue treatment during conception, pregnancy, and breastfeeding [43]. The relationship between IL-23 biology and pregnancy outcomes, including pre-eclampsia, remains incompletely understood, and the clinical implications of IL-23 inhibition in this context are uncertain [44,45]. Further prospective data are awaited from the ongoing PIANO registry, which is evaluating pregnancy outcomes among patients with IBD exposed to guselkumab [46].

## 6. Real-World Experience

Randomised controlled trials remain the cornerstone for evaluating efficacy and safety, but their strict eligibility criteria may limit generalisability to routine clinical practice. A retrospective analysis reported that only 16.9% of patients encountered in routine IBD practice would have met the eligibility criteria for the corresponding pivotal clinical trials, falling to 8% for more contemporary studies [47,48]. These findings highlight the importance of complementary real-world evidence to better understand treatment effectiveness across the broader IBD population.

Real-world experience with guselkumab in IBD remains limited. For ulcerative colitis, a single-centre US cohort included 61 patients, of whom 30 had active disease at treatment initiation [49]. At Week 12, clinical remission was achieved in 83% of patients with active disease, while median faecal calprotectin decreased from 977 μg/g to 322 μg/g. Remission rates were similar in patients with and without prior ustekinumab exposure, although the number of ustekinumab-exposed patients was small (*n* = 14). It is important to note that the findings of this study were only available as a published conference abstract and should be regarded as preliminary [49].

Similarly, early real-world experience in Crohn’s disease has been reported from a prospective single-centre cohort that included 77 patients with moderately-to-severely active disease treated with guselkumab for at least 12 weeks [50]. This represented a highly treatment-refractory population, with 58% having undergone previous intestinal surgery and 69% exposed to two or more prior advanced therapies. By Week 12, the median Harvey–Bradshaw Index value improved from 8 to 6.5, steroid-free remission increased from 56.6% to 68.6%, median C-reactive protein decreased from 8 mg/L to <3 mg/L, and 60% of patients achieved faecal calprotectin remission (<150 μg/g). No treatment-related adverse events were reported. It is important to note that the findings of this study were only available as a published conference abstract and should be regarded as preliminary [50].

Data evaluating intra-class switching between IL-23p19 inhibitors remain limited. In a real-world cohort of 28 patients with Crohn’s disease refractory to previous risankizumab treatment, 89% had received at least three prior advanced therapies and 89.3% had experienced primary or secondary loss of response to risankizumab. Following treatment with guselkumab, steroid-free remission increased from 35.7% at baseline to 68.2% at Week 12, accompanied by significant improvements in the Harvey–Bradshaw Index (median reduction five points) and faecal calprotectin (median reduction 488 μg/g), with no new safety concerns identified. It is important to note that the findings of this study were only available as a published conference abstract and should be regarded as preliminary [51].

Several prospective real-world studies are now underway. The GORGEOUS study is evaluating the effectiveness and safety of guselkumab in routine clinical practice across Germany, including in predefined patient subgroup [52]. Similarly, the UK-based GUSTO-UK study is recruiting participants to evaluate the effectiveness of guselkumab across a range of clinical phenotypes and should provide important insights into its real-world performance [53].

## 7. Practical Considerations and Treatment Positioning

**Figure 3:**
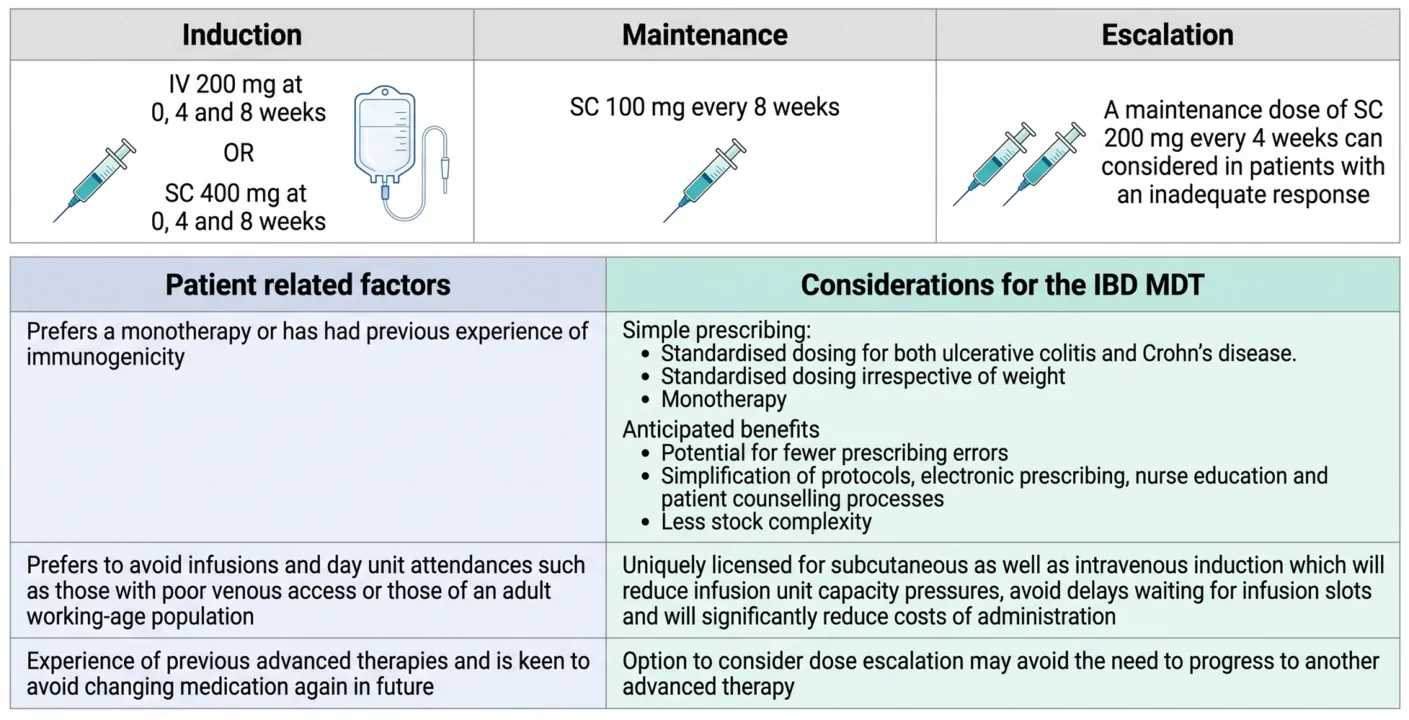
Summary of the practical considerations for prescribing guselkumab for IBD, including the dosing guidance and specific reference to the relevant patient-related factors as well as considerations for the wider IBD MDT. Created using https://BioRender.com.

Practical differences between IL-23p19 inhibitors may influence treatment selection in routine clinical practice. Unlike risankizumab and mirikizumab, which require intravenous induction before transitioning to subcutaneous (SC) maintenance, guselkumab is the only IL-23p19 inhibitor licensed with the option of either intravenous or fully SC induction for both ulcerative colitis and Crohn’s disease. Avoiding intravenous induction has the potential to be more convenient for patients. Specific patient-related factors may also favour a subcutaneous induction approach, including those with poor venous access, rural populations and working-age adults (Figure 3).

An additional practical advantage is that the licensed induction and maintenance schedules are identical across ulcerative colitis and Crohn’s disease, simplifying prescribing and reducing the potential for dosing errors. Guselkumab is available as pre-filled SC injection devices (PushPen and One-Press), allowing self-administration following appropriate patient education (Figure 3).

Unlike risankizumab and mirikizumab, guselkumab also offers two licensed maintenance dosing strategies for Crohn’s disease (100 mg every 8 weeks or 200 mg every 4 weeks), providing an opportunity to individualise treatment. Post hoc analyses of the pooled GALAXI-2 and GALAXI-3 trials suggested that patients with a greater inflammatory burden (CDAI > 300, SES-CD > 12, or C-reactive protein > 5 mg/L) derived greater benefit from the higher maintenance dose, whereas outcomes were similar between regimens in patients with less severe disease [15]. However, prospective comparative and cost-effectiveness studies are needed before baseline dose selection based on inflammatory burden can be recommended.

Dose optimisation may also be considered during follow-up. In the QUASAR long-term extension study, participants with an inadequate response who underwent maintenance dose escalation demonstrated improvements in both clinical response and clinical remission without new safety concerns, although interpretation is limited by the relatively small number of patients undergoing dose adjustment [22]. These findings provide early support for maintenance dose optimisation where clinically indicated. Whether these flexible dosing strategies improve long-term outcomes or represent a cost-effective approach remains uncertain. Further real-world evidence will be important, particularly in patients previously exposed to ustekinumab or another IL-23 inhibitor who were under-represented in the registrational trials (Figure 3).

In current practice, several features may make guselkumab an attractive treatment option for selected patients. The availability of a fully subcutaneous induction regimen offers greater convenience for patients wishing to avoid intravenous therapy and may reduce demands on infusion services. Flexible maintenance dosing also allows treatment to be tailored according to disease severity, with the higher 200 mg every 4 weeks regimen providing an option for patients with a high inflammatory burden or more aggressive Crohn’s disease. Emerging evidence also suggests promising efficacy in fistulising perianal Crohn’s disease, an area of considerable unmet clinical need, although confirmation from the peer-reviewed publication of the FUZION study is awaited. Guselkumab may also be particularly attractive for patients with co-existing psoriasis or psoriatic arthritis, where it has established efficacy and regulatory approval. Although direct head-to-head evidence is lacking, there were improved endoscopic outcomes observed with guselkumab compared with ustekinumab in the GALAXI programme, although it is important to emphasise that the study was not powered for formal superiority testing. However, these results, together with the results of the SEQUENCE trial supporting selective IL-23 inhibition over IL-12/23 blockade, have highlighted this as an important area to direct future research. Ultimately, treatment choice will continue to be influenced by local prescribing pathways, healthcare costs, previous biologic exposure, and the availability of lower-cost biosimilars. As comparative studies and real-world experience expand, clinicians will be better placed to define the optimal role of guselkumab within the treatment pathway for IBD.

## 8. Conclusions

Guselkumab is an important addition to the expanding IL-23p19 inhibitor class for IBD. Across phase III programmes in Crohn’s disease and ulcerative colitis, there has been a suggestion of efficacy across clinical, endoscopic and histological endpoints, together with a favourable short-to-medium-term safety profile and low immunogenicity. Practical advantages include the option of fully subcutaneous induction, identical dosing schedules for both Crohn’s disease and ulcerative colitis, and maintenance dose escalation as an option for selected patients. Emerging data in fistulising perianal Crohn’s disease and extraintestinal manifestations also suggest potential benefit in patient groups with substantial unmet need.

Several important questions remain. Longer-term IBD-specific safety data, real-world studies, cost-effectiveness analyses, and direct comparisons with other IL-23p19 inhibitors are still needed. Combination advanced therapy is another area of interest. The VEGA study showed improved efficacy with combined guselkumab and golimumab compared with either treatment alone, without an increase in serious adverse events, and the ongoing DUET-UC/ENCORE-UC and DUET-CD/ENCORE-CD studies will help determine whether this approach has a role in routine practice. Finally, the IL-23 pathway continues to evolve beyond monoclonal antibodies, with oral IL-23 receptor antagonists such as icotrokinra offering another potential therapeutic approach [54]. Overall, guselkumab is a valuable addition to the IBD treatment armamentarium, and its place in clinical practice will become clearer as further evidence emerges.

## Figures and Tables

**Table 1:** Comparison of IL-23p19 inhibitors approved for inflammatory bowel disease.

Name	Licensed Indications (MHRA)	Pharmacological Characteristics	Induction Dosing	Maintenance Dosing
Guselkumab (Tremfya)	Plaque psoriasis (2017) Psoriatic arthritis (2020) CD (2025) UC (2025)	Fully human IgG1λ monoclonal antibody with a native Fc domain capable of binding FcγRI (CD64); the clinical relevance of this interaction remains uncertain. Low risk of immunogenicity.	CD & UC: 200 mg IV at Weeks 0, 4 and 8 or 400 mg SC at Weeks 0, 4 and 8	CD & UC: 100 mg SC every 8 weeks or 200 mg SC every 4 weeks
Risankizumab (Skyrizi)	Plaque psoriasis (2017) Psoriatic arthritis (2021) CD (2023) UC (2024)	Humanised IgG1 monoclonal antibody containing LALA Fc mutations that minimise Fcγ receptor binding and Fc-mediated effector functions.	CD: 600 mg IV at Weeks 0, 4 and 8 UC: 1200 mg IV at Weeks 0, 4 and 8	CD & UC: 360 mg SC every 8 weeks
Mirikizumab (Omvoh)	UC (2023) CD (2025)	Humanised IgG4 monoclonal antibody containing FALA Fc mutations that minimise Fcγ receptor binding and Fc-mediated effector functions.	CD: 900 mg IV at Weeks 0, 4 and 8 UC: 300 mg IV at Weeks 0, 4 and 8	CD: 300 mg SC every 4 weeks UC: 200 mg SC every 4 weeks

Abbreviations: CD, Crohn’s disease; FcγRI, Fc gamma receptor I; IgG, immunoglobulin G; IV, intravenous; MHRA, Medicines and Healthcare products Regulatory Agency; SC, subcutaneous; UC, ulcerative colitis.

**Table 2:** Registrational (phase III) clinical trials of guselkumab for moderate-to-severe UC.

Trial	Trial Design	Population Characteristics	*n*	Duration (Weeks)	Primary Outcome	Key Safety Findings	Key Efficacy Findings
QUASAR	Induction: randomised (3:2), placebo-controlled; guselkumab 200 mg IV at Weeks 0, 4 and 8	Adults (mean age 41 years) Moderate-to-severe UC 48% biologic/JAK inhibitor-naïve	701	12	Clinical remission at Week 12 ^†^	At Week 12: adverse events were reported for 49% of patients treated with guselkumab and 49% of placebo-treated patients and serious adverse events for 3% and 7%, respectively.	Clinical remission: 22.6% vs. 7.9% (*p* ≤ 0.001)
	Maintenance: Week 12 responders randomised (1:1:1) to guselkumab 100 mg SC every 8 weeks, guselkumab 200 mg SC every 4 weeks, or placebo	Adults (mean age 41 years) Moderate-to-severe UC 42% biologic/JAK inhibitor-naïve	568	44	Clinical remission at Week 44 ^†^	At Week 44: the proportions of adverse events was similar across treatment groups with 65% in the 100 mg group, 70% in the 200 mg group and 68% in the placebo group.	Clinical remission: 45.2% (100 mg q8w) and 50.0% (200 mg q4w) vs. 18.9% placebo (*p* ≤ 0.001)
ASTRO	Treat-through: randomised (1:1:1), placebo-controlled; guselkumab 400 mg SC at Weeks 0, 4 and 8 followed by 100 mg SC every 8 weeks or 200 mg SC every 4 weeks, or matched placebo	Adults (mean age 42 years) Moderate-to-severe UC 58% biologic-, JAK inhibitor- and S1P modulator-naïve	418	24	Clinical remission at Week 12 ^†^	At Week 24: the proportions of adverse events and serious adverse events were similar across groups and reported as 53% in the 400/100 mg group, 61% in the 400/200 mg group and 65% in the placebo group.	Week 12: clinical remission 28% vs. 6% (*p* ≤ 0.001) Week 24: clinical remission 35% (100 mg q8w) and 36% (200 mg q4w) vs. 9% placebo (*p* ≤ 0.001)

^†^ Clinical remission defined as Mayo stool frequency subscore of 0 or 1 (not increased from baseline), rectal bleeding subscore of 0, and Mayo endoscopic subscore of 0 or 1 without friability. Abbreviations: JAK, Janus kinase; SC, subcutaneous; S1P, sphingosine-1-phosphate; UC, ulcerative colitis.

**Table 3:** Registrational (phase III) clinical trials of guselkumab for moderate-to-severe CD.

Trial	Trial Design	Population Characteristics	*n*	Duration (Weeks)	Co-Primary Endpoints	Key Safety Findings	Key Efficacy Findings
GALAXI-2	Induction & Maintenance: Identically designed, randomised, double-blind, treat-through trial. Participants were assigned (2:2:2:1) to: Guselkumab 200 mg IV → 200 mg SC q4w Guselkumab 200 mg IV → 100 mg SC q8w Ustekinumab Placebo Placebo non-responders received masked ustekinumab rescue from Week 12.	Adults (mean age 37 years) Moderate-to-severe CD 41.9% biologic-naïve	508	48	Clinical remission (CDAI < 150) and endoscopic response ^†^ at Week 48	At Week 48: the proportion of adverse events in the pooled GALAXI-2 and GALAXI-3 data set were confirmed as 76% in the guselkumab 100 mg group, 79% in the ustekinumab group and 54% in the placebo group	Week 48 Clinical remission: 55% (200 mg q4w) 49% (100 mg q8w) 12% placebo (*p* < 0.0001) Endoscopic response: 38% (200 mg q4w) 39% (100 mg q8w) 5% placebo (*p* < 0.0001)
GALAXI-3	Identical design to GALAXI-2.	Adults (mean age 37 years) Moderate-to-severe CD 41.5% biologic-naïve	513	48	Clinical remission (CDAI < 150) and endoscopic response ^†^ at Week 48	At Week 48: the proportion of adverse events in the pooled GALAXI-2 and GALAXI-3 data set were confirmed as 76% in the guselkumab 100 mg group, 79% in the ustekinumab group and 54% in the placebo group	Week 48 Clinical remission: 48% (200 mg q4w) 47% (100 mg q8w) 13% placebo (*p* < 0.0001) Endoscopic response: 36% (200 mg q4w) 34% (100 mg q8w) 6% placebo (*p* < 0.0001)
GRAVITI	Induction & Maintenance: Randomised (1:1:1), placebo-controlled, treat-through trial. Guselkumab 400 mg SC → 100 mg SC q8w Guselkumab 400 mg SC → 200 mg SC q4w Placebo Placebo participants meeting rescue criteria received guselkumab from Week 16.	Adults (mean age 38 years) Moderate-to-severe CD 54% biologic-naïve	347	48	Clinical remission (CDAI < 150) and endoscopic response ^‡^ at Week 12	At Week 48: the proportion of adverse events was similar across treatment groups at 13% in the 400 mg/100 mg group, 8% in the 200 mg group and 14% in the placebo group	Week 12 Clinical remission: 56.1% vs. 21.4% placebo (*p* ≤ 0.001) Endoscopic response: 41.3% vs. 21.4% placebo (*p* ≤ 0.001) Week 48 Clinical remission: 66.1% (200 mg q4w) 60.0% (100 mg q8w) 17.1% placebo (*p* ≤ 0.001) Endoscopic response: 51.3% (200 mg q4w) 44.3% (100 mg q8w) 6.8% placebo (*p* ≤ 0.001)

^†^ Co-primary endpoints: clinical response at Week 12 with (1) clinical remission or (2) endoscopic response at Week 48. Clinical remission was defined as CDAI < 150. Endoscopic response was defined as ≥50% reduction from baseline SES-CD or SES-CD ≤ 2 in participants with isolated ileal disease. ^‡^ Co-primary endpoints: clinical remission and endoscopic response at Week 12. Clinical remission was defined as CDAI < 150. Endoscopic response was defined as ≥50% reduction from baseline SES-CD. Abbreviations: CDAI, Crohn’s Disease Activity Index; CD, Crohn’s disease; IV, intravenous; q4w, every 4 weeks; q8w, every 8 weeks; SC, subcutaneous.

## Data Availability

No new data were created or analyzed in this study. Data sharing is not applicable to this article.

## Abbreviations

Anti-TNFα, anti-tumour necrosis factor alpha; BMI, body mass index; CD, Crohn’s disease; CDAI, Crohn’s Disease Activity Index; CRP, C-reactive protein; EMA, European Medicines Agency; EIM, extraintestinal manifestation; Fc, fragment crystallisable; FcγRI, Fc gamma receptor I; FDA, US Food and Drug Administration; HBI, Harvey–Bradshaw Index; HuCAL, Human Combinatorial Antibody Library; IBD, inflammatory bowel disease; IgG, immunoglobulin G; IL, interleukin; IL-23R, interleukin-23 receptor; IV, intravenous; JAK, Janus kinase; MHRA, Medicines and Healthcare products Regulatory Agency; MRI, magnetic resonance imaging; q4w, every 4 weeks; q8w, every 8 weeks; RCT, randomised controlled trial; SAE, serious adverse event; SC, subcutaneous; SES-CD, Simple Endoscopic Score for Crohn’s Disease; S1P, sphingosine-1-phosphate; UC, ulcerative colitis.
